# Virtual oceans: how VR technologies mediate oceanic space

**DOI:** 10.1007/s40656-025-00714-1

**Published:** 2026-01-13

**Authors:** Jesse Peterson

**Affiliations:** Radical Humanities Laboratory, Department of Geography, University College Cork, Cork, Ireland

**Keywords:** Virtual reality, Marine science, Geography, Oceans, Technological mediation

## Abstract

In marine science, VR technologies are being used to model underwater space and enable virtual geoscience fieldtrips for teaching and research. The vast potential in adapting these technologies alongside their speedy application suggests challenges in relation to the standardization of these technologies and what forms of representation come to matter in these contexts. This raises the question regarding how the use of VR technologies produce and transfer knowledge about marine environments. To address this question, I explore VR technologies as tools for mediating human-ocean relations, analyzing processes and technologies used in marine science to produce VR models and digital environments of oceanic spaces that give meaning to the marine. Doing so, I argue that VR technologies flatten the vast materiality of the oceans to create an illusion of depth that is anchored in the “objectivity” of the visual. Additionally, VR oceans currently represent a shift from other representations of the oceans as global or planetary, as they are being used to assist the production of local, place-based engagements of the seas, specifically through positionality and spatial awareness (aka proprioception), which differs from previous representations of the sea based on sight.

## Marine science and virtual reality technologies

The interests of marine scientists into the qualities and contents of the oceans guide the development of specific technologies – such as maps and satellite imagery – to provide the means for understanding them. However, in-depth study into some of these different technologies from critical historical and social perspectives are few and far between. Though remote sensing has emerged as a topic of interest, new studies on data generation, data processing, AI and machine learning, and autonomous sensor systems are required. For instance, recent technological advances in digital photography, drones (e.g., remotely operated vehicles and automated underwater vehicles), and computer science are contributing to further changes in the way oceanic space is understood and researched.

This article contributes to this body of research by looking in-depth into the uses of virtual reality (VR) technologies that are becoming more commonplace in scientific and artistic research methods, as tools for data collection and as vehicles for digitized representations (Matthews, 2018). For the purposes of this article, I define VR technologies as a wide range of technologies (oftentimes dependent upon other technologies) used to produce spectacle and frame viewers within it for the purposes of capturing and virtually simulating sense experience. In the field of marine science, for instance, VR technologies appear to promise easier access to the seas in an “immersive” way. This promise of immersion can be observed in the development of VR devices to be used underwater (Costa et al., 2017) as well as their applications. VR technologies have been or are being used to visually model underwater space and objects (Bellarbi et al., 2013; Palmese & Trucco, 2008), assist remote operations of diving vessels (Fleischer et al., 1995; Lin & Kuo, 1998), analyze animal behavior (Butail et al., 2012), allow for simultaneous collaborations through telepresence (Raineault et al., 2018), and enable virtual geoscience fieldtrips for research, policy, and education (Fröhlich, 2000; Merchant et al., 2014; Newell et al., 2017). Across the field, VR technologies are being used to assist and offer improved intimacy and integration between human and ocean for practical, pedagogical, and analytic reasons.

Nonetheless, the vast potential in adapting these technologies alongside their speedy application suggests challenges in relation to the standardization of these technologies, the contexts they are used in, and what forms of VR representations come to matter. Hence, I explore how the use of VR technologies produce and transfer knowledge about marine environments. Specifically, how is oceanic space produced through VR technologies, how does VR mediate oceans in marine science, and what practices does such mediation help constitute?

To address these questions, I first discuss how VR technologies function as forms of mediation in the next section. Following that, I then present interrelations between scientists and their instruments – using an interdisciplinary approach, drawing primarily on geography and science and technology studies – enquiring into the practices that make VR oceans, the VR technologies used, and the digital spectacles produced. Specifically, I analyse how multiple technologies are put to use through a 3-step process of data generation and selection, 3D model building, and the ensuing representations of VR oceans. Through this analysis, I demonstrate how the use of VR technologies in marine science construct oceanic space as scientific products and how these products inform scientists and their object of study. Following this analysis, I then discuss how VR technologies mediate scientists with the oceans while simultaneously producing oceans as media. From this, I argue that VR technologies offer a unique form of sensory mediation upon oceanic space not yet delivered through other technologies on their own, highlighting that VR technologies cannot duplicate the ocean. Instead they add another technological layer of mediation within marine sciences, which currently represents a (re)turn towards oceans at local scales, through emphasis on volume and verticality and sense ‘immersion.’

## VR technologies as forms of mediation

Across the social sciences and humanities, technologies have been understood as mediating humans to and with the environment. Not only do technologies couple humans to environments, but human values, histories, and culture are physically manifest in technologies themselves (Leroi-Gourhan, 1993; Kittler, 1999). Hence, technologies have been understood as prostheses that augment human senses and abilities, impacting culture and the human mind (McLuhan, 1994) and offering new forms of mutually reinforcing intimacy (Fish, 2024, p. 6). Technologies are always embedded in bodily relations (Ihde, 2002), or, even more provocative, may even be an original condition of human bodily development and cognition (Coté, 2010).

Put somewhat differently, society engages in forms of technological mediation in which specific technologies impact and transform how humans perceive, communicate about, and engage with the world (Rosenberger & Fried, 2021, pp. 3–4). For instance, in relation to Western science, technologies have been understood to assist scientists to “mediate” natural phenomena by making these *legible* (e.g., “inscription devices”, see Latour & Woolgar, 2013). To do so, technologies function as tools that mediate natural phenomena by *making* media (e.g., charts, maps, photographs) but also as active instruments *mediating* the relations between scientists and their object(s) of study (de Boer et al., 2021, p. 394). In other words, one cannot understand the world without understanding the people and technologies used to interact with it (Russo, 2022).

Since the quest for knowing the deep oceans “requires the mediation of science and technology” and comes about “only through chains of mediation and remote sensing” (Alaimo, 2019, p. 429; Jue, 2020, p. 3), it becomes significant to understand the relations between scientist, ocean, and technology. Two major implications arise from this observation. First, scientists interpret their object of study through the tools at their disposal. These tools impact how the object gets interpreted by the scientist. In this way, technologies assist in creating scientific practices that stem from “asymmetrical relations between ‘interpreter’ and ‘interpreted,’” which stresses how power discrepancies can influence the “character” of the relations between scientist and study object (de Boer et al., 2021, pp. 398–399). Second, technologies also may function as “environing media.” For instance, technologies—including maps, chronometers, floating buoys, satellites, and more—mediate the oceans through “gathering, processing and disseminating environmental data and information” and “condition [human] understanding of what the ocean is, how it changes and what is considered essential and ‘actionable’ ocean knowledge” (Lidström et al., 2023, pp. 114–115). In other words, technologies both make aspects of the oceans comprehensible to human understanding while simultaneously helping to produce a particular kind of environment to which humans can respond and alter if they so choose. Because technologies and the models they make assist in shaping environments and human modes of engagement with them rather than merely representing them (Boon & Knuuttila, 2009), it becomes of scholarly interest to inquire how VR technologies mediate the seas.

Thus, by understanding VR technologies as a form of technological mediation between oceans and humans, this article takes an interdisciplinary approach to address what kinds of oceans become possible and come into being through such technologies. In the following section, I present a short, overview of technologies that have produced “virtual” realities of the oceans, followed by an analysis of the use of VR in marine science. By conceptualizing VR technologies as actively mediating the ocean means that I attend to the ongoing human-nonhuman relations that occur in the implementation of VR technologies in marine science. In so doing, I analyze practices employed by scientists with VR technologies to demonstrate “knowledge production as situated within new technical domains and new political contexts” (Stephens & Lewis, 2017). Also, I perform critical analysis on the technologies and practices that assist in producing scientific objects, specifically by attending to the ways they assist scientific production for identifying any existing internal politics (Pickett et al., 2020). Finally, I rely upon informal interactions and conversations with scientists as well as the media produced (e.g., publications, photographs, and models) in their pursuit of building oceans in virtual reality (see Doel & Henson, 2006) to best understand how these tools mediate the seas. Through this analysis of practices and technologies in context and what they produce, this article explores ways by which VR technologies mediate oceanic space and its implications for human-ocean relations.

## Making oceanic spaces virtual

### Virtual (techno)realities of the oceans

Virtual oceans have been created through various methods and technologies that have been used throughout human history to better sense the vast scale and watery, pressurized depths of the oceans (see Deacon, 2018). Scholars working amidst the intersections of philosophy, history, marine and social sciences have described and analyzed multiple contexts, stories, and concepts that undergird several of these technologies. The construction of the ocean dovetailed with building empires and advancing science theoretically and methodologically in the 19th century (Reidy & Rozwadoski, 2014). Historians like Helen M. Rozwadoski and Naomi Oreskes have provided detailed insights into the working lives, tools, and techniques of marine scientists who used sounders, trawlers, and submersibles in their quest to acquaint themselves with the deep ocean through precision measurements and direct experience (Rozwadoski, 2005; Oreskes 2021). Maps, such as the Heezen-Tharp mapping project, have also been instrumental in visualizing the seabed as anti-submarine warfare and transcontinental telecommunications became central concerns during the Cold War (Doel et al., 2006). From such work, one learns that marine scientists and their technologies have relied on and taken advantage of military and commercial interests (Oreskes, 2003, 2022; Doel et al., 2006) to further scientific understandings of the deep. As a result, interests in first-hand experience, data capture and oceanic representation through technological means permeate many of the modern methods and tools for understanding and studying the oceanic depths, including acoustic-sounding technologies like sound navigation and ranging (i.e., SONAR) (Katzir, 2023), water-penetrating color film, and airborne lidar bathymetry (Makowski & Finkl, 2016, pp. 16, 23–25).

Today, scientists studying the deep environs of the oceans and seas still rely on techniques and technologies developed over the last 100 years to recreate oceans virtually. Given that light (or electromagnetic radiation) does not penetrate seawater, many modern technologies rely on acoustics to gather information. For instance, SONAR uses pulses of acoustics energy, referred to as “pings,” to travel from a source (e.g., transducer) towards the seafloor and then back. The time for the ping to travel to and from the source helps to solve for ocean depth (e.g., bathymetry), while the amount of the signal that returns (i.e., signal strength) assists in interpreting the composition of the seafloor (e.g., backscatter), and structure of the sub-seabed (e.g., sub-bottom profiles). Acoustic-based remote sensing technologies include sidescan SONAR and echo sounder reflection (e.g., single beam, multibeam, and seismic). Additionally, technologies capturing visual information, including aerial photography, orthoimagery, airborne laser bathymetry, and satellite imagery, have all been used to map the seafloor. Remote operated vehicles mounted with cameras are also being used to acquire video and image data. As a result, such technologies promise “vicariate access to unknown realms” (Makowski & Finkl, 2016, p. 46), such as the oceans.

Last, increasing availability of consumer VR technologies promise a deepening of “access” to the oceans as they appropriate oceanic information provided through these and other technologies. Though innovations in visual “immersion” begin as early as the late 18th century, such as with the patenting of Robert Barker’s panorama (see Trumpener & Barringer, 2020) – a 360-perspective artwork installation where the viewers are brought “within” the frame rather than positioned outside it – the main understanding of VR imaging technologies as a method to simulate sense experience virtually begins in the mid-20th century (Gigante, 1993). From Heilig’s Sensorama (built to simulate the experience of riding a motorcycle) to the development of VR headsets, tracking cameras, and haptic feedback devices (Berkman, 2024), technological products and innovations in commercial VR technologies attempt to immerse users in virtual environments with which they can interact and become involved (Mazuryk & Gervautz, 1996; Lum et al., 2020). In this way, consumer VR technologies rely upon the use of a range of other technologies that produce the oceans as virtual. Indeed, scientists must gather data, simulate data, render the output, and display it (Wheless et al., 1995, p. 53). The labor involved takes places in various locales through multi-sited mediations, specifically in the field, in the computer lab, and then through an applicable output device. When used all together, these VR technologies add another layer to how the ocean gets mediated; ultimately offering a stereoscopic image that changes dynamically with a user’s perspective along with the potential for additional audio, haptic, and sensory feedback mechanisms. Since marine science has incorporated digital components to move from studying the deep seas in person to also being able to do so using remote operations (Höhler, 2002), VR technologies offer a compelling form of mediation that promises to bring these remote forms of access back into a personal realm, albeit one that takes place in the virtual.

### Flattening the field

Producing VR environments entails countless negotiations and compromises, involving the technologies and environs themselves, the scientists’ understanding of them, how such technologies get used, and the data they capture.

**Fig. 1:**
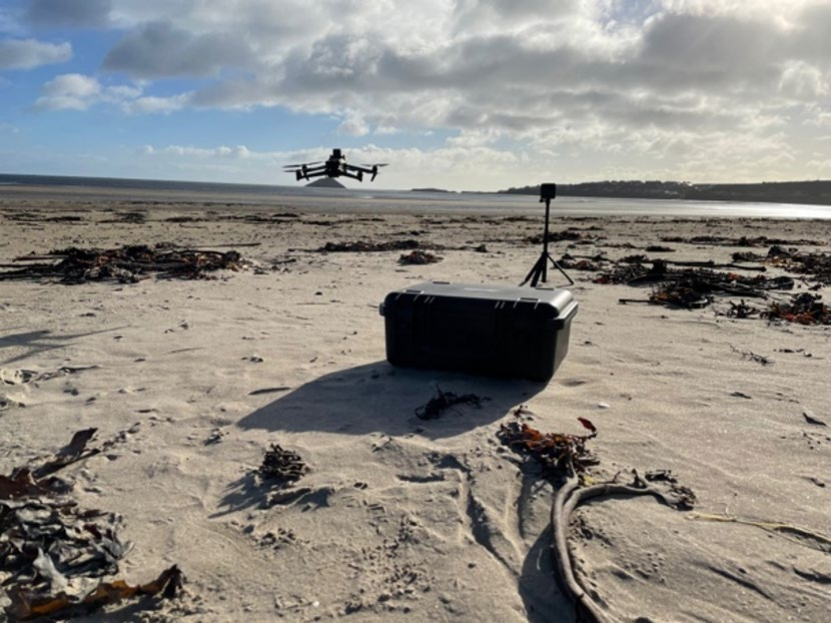
Field site visit using an aerial drone to visually map a local coast. Photo by author

As a result, oceanic space ends up simplified and flattened in the early stages of producing VR oceans. The tools to construct VR oceans need be accessible and are designed for capturing data in specific ways. For instance, during a trial run to develop a VR environment of nearby coastline in southern Ireland, I assisted in piloting a drone to capture digital photographs of the area (see Fig. 1). This drone was accessible for use because of successful grant capture, which provided the means to purchase it. However, we needed to use the drone from the coastline because we did not have access to a boat on this day.^1^ The drone served as a navigable platform for taking digital photographs and videography, which flattened the coast, its grit and wet, salty aromas, and weight, into 2D imagery made up of pixels. Moreover, we needed to make sure that weather conditions were sufficient, specifically that it was not too windy or wet, and that the drone would be operable. Upon arrival, we needed to make decisions and adjust our plans based on concerns over environmental and social factors, such as the presence of birds, battery charge, and possible disturbance to other humans on site, which assisted in determining the location, route, and area covered. We also needed to make necessary preparations to install the batteries, assemble the drone, and mount the positioning system correctly. After all this, we tried to fly the drone but soon discovered that the controller was unable to geolocate it correctly. After some troubleshooting, we set the flight route and interval for taking photographs and proceeded to send the drone on its flight path. The drone navigated automatically for about 10 min before finishing, after which we set about taking some multispectral photographs of the site using the drone manually, capturing ultraviolet and near infrared bands. In this process, none of the participants (including myself) required any special expertise to fly the drone and take photographs. However, we needed some operational knowledge about setup and setting the route and proper parameters. Moreover, we needed the acumen to operate the drone in ways that responded to the environmental and social context. Even though the drone literally made 2D representations of the place by taking photographs, this technology also “flattened” our experience of the place, as we worked to protect its use for the future, while simultaneously focusing our attention on other aspects of this place we may not have considered otherwise (see Fish, 2019).

If we had been able to work underwater that day, we would have needed to achieve a balance between manual oversight and allowing for automated data capture amidst the need to deal with the ocean’s elementality (Fish, 2024, 178–180). Because the ocean asserts itself in unexpected ways. The physical characteristics of the ocean provide the means for monitoring it while simultaneously acting as the prime hurdle, visible in how drones and UAVs are designed and their use get finessed to parse through the pressure, salt, sediment and debris, animal life, currents, and low lighting in this place. In other words, the greater contexts of science in action, technological materialities, scheduling, expense, and the elementality of the location under observation all assert themselves in the process of producing VR oceans, while often remaining backgrounded in the final product.

**Fig. 2:**
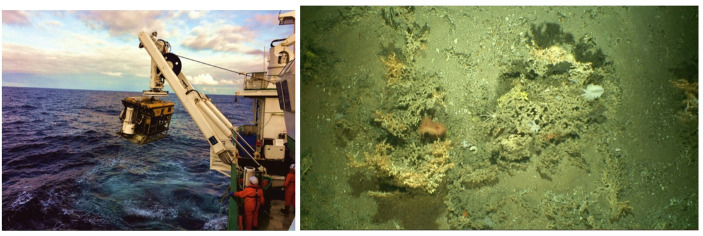
**a** The Holland I ROV being deployed off the side of the RV Celtic Explorer for capturing an array of data on cold water corals. Photo by Zoe Lim.** b** Example photograph of a ‘clean’ image for making a VR representation of underwater coral

The multiple compromises and negotiations made to capture this data, therefore, often requires methods for dealing with these contexts and the oceans themselves, specifically standardization, automation and bulk capture. For instance, pilots navigate drones underwater across a chosen area at a speed of approximately 0.4 knots and at about 1.5 m above the seafloor. During this time, the drone’s positioning system records its location, while its lights (up to 12 with varying wattages [250–400]) illuminate the surrounding space. The velocity of the drone and its distance from what it is recording provide a standardized perspective from which video gets recorded (e.g., the field of view remains constant) as well as prevents the thrusters of the remotely operated vehicle (ROV) from resuspending sediment into the water column and thus blocking the field of view (Fig. 2). Additionally, several lights are required to ensure that the seabed is homogenously lit (Lim et al., 2020). While the drone is deployed, scientists sit onboard a ship, instruct pilots where to navigate the drone (which can go down to > 1000 m water depth), and record their observations in a scientific log. Video, photographs, and other data gathered by the drone during its trip get sent to the ship through a fibre optic cable and are stored and backed up for inspection and subsequent onshore data processing in the lab. To deal with the sea’s materiality, strict protocols and methods must be followed. Doing so standardizes the process for taking photographs, while also limiting how the technologies might get used otherwise.

Even though such standardizations may help streamline the process, bulk capture is also required to ensure enough usable data gets produced. For instance, the drone’s pressurized HD camera is set up to record the ocean at up to 50 frames per second (fps), which is approximately double the frame rate for traditional motion capture (24 fps) and varies depending on data quality and velocity of the ROV at the time of acquisition. This high frame rate ensures a large volume of data. In fact, ROV dives may acquire video data continuously for over 24 h, with potentially hundreds of dives taking place per offshore expedition. Doing so demonstrates that VR mediations of the ocean begin in an emphasis on precision and quantity to deal with the challenges that the oceanic environment presents. Through bulk capture and standardization, the first stage in producing VR oceans must sideline a great deal of the ocean’s very essence, flattening it in the process.

### Adding depths

Through standardization, bulk capture, and automation, scientists flatten the sea. However, counterintuitively, this process also provides the foundation to replicate its depth. In principle, one needs only two photographs taken from slightly different directions, as in stereoscopic imagery, to produce a sense of depth. However, to create 3D models and VR environments, technologies that can process and “clean” large volumes of data are necessary. With underwater video, scientists watch the videos and choose specific time periods of interest from which they extract video stills, which serve the same function as digital photographs. To extract stills, scientists use 3D computer graphics software (e.g., Blender) that can extract frames from videos at a specific rate (e.g. 2 frames every 10 s) and for a set time segment, usually a part of the film that captures cold-water coral reefs, shipwrecks, benthic habitats or bedrock occurrences (de Oliveira et al., 2022). Through this process, the number of images extracted can be over 10,000 in total. With so many stills, marine scientists further reduce the number of images they will use to build the 3D model. They must remove any blurry images as any “dud” image will disrupt the rendering process. Additionally, they may further refine this selection based on predetermined filters, such as brightness, contrast, or high levels on the red channel, as shorter light wavelengths attenuate the most in underwater conditions leading to poorer quality images with higher color distortion and lower contrast (Galdran et al., 2015). This process can take months, as camera focus, backscatter (e.g., suspended debris in the water column), or anything moving (e.g., fish) in a single image can affect the successful production of a 3D model. Scientists, therefore, require a very specific kind of photograph to enable the construction of 3D models, essentially getting rid of images that depict anything of or in the sea that interrupts the view. As with the drones, filtering data consists of greater reliance upon automatic methods for achieving a usable data set.

**Fig. 3:**
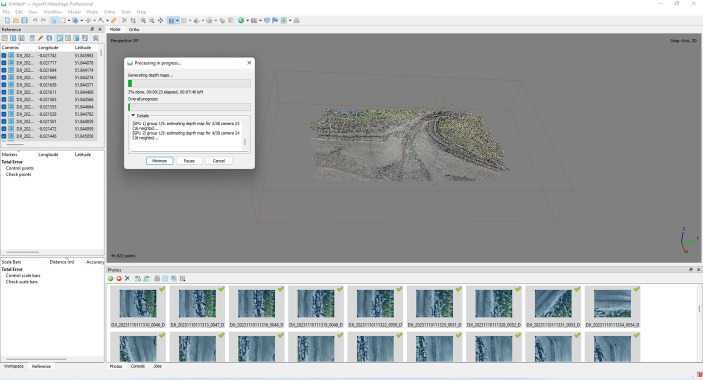
Point cloud reconstruction from drone footage taken during our field site visit to Irish coast

By homogenizing the stills, scientists can then produce 3D models of the sea through photogrammetry. However, they do not replicate a fixed ocean environment but rather make modular and pliable digital representations of the sea. Through photogrammetry software, scientists develop their stills into 3D models through various stages, including the identification of common features in overlapping images (Lowe, 2004; Massot-Campos & Oliver-Codina, 2015) and Structure-from-Motion (SfM) which plots these commonalities in a 3D space as points and which is called a ‘point cloud’ (Lim et al., 2020) (Fig. 3). Point clouds can then be converted to a topographical surface called a mesh, and upon which the 2D images can be overlaid to provide the model with depth but also “texture.” Because this process must make angle corrections through orthorectification of the raw images, the 2D images overlaid upon the mesh will be distorted (e.g., compressed or stretched) to account for the artificial addition of depth. These concerns, albeit interesting, are not of supreme importance to the scientist, because concern involves the resolution or saturation of the point cloud, aiming to produce a model which contains not too many but not too few points in the model. The priority is on the structure and not on how it looks. Ultimately, this process does not produce an exact 3D replica, but an idealized duplicate based on mathematical averages. This averaged 3D model thus serves as scaffolding, a tabula rasa, or blank slate, upon which scientists can attach qualifying data.

In other words, the desired point cloud depends on the research question, meaning that scientific intention is important for what scaffolding gets chosen and what it comes to mean. For instance, 3D models produced through photogrammetry can be represented as point clouds, meshes, orthophotographs or digital elevation models. These digital amalgamations, however, do not mean until analyzed. Oceanic space in the 3D models gets configured through their interpretation and analysis. For instance, point clouds become useful because they can be classified and interpreted in multiple ways (Rosenberger, 2011), such as through object-based image analysis. In this method, the models must be segmented into different units. Each voxel (a 3D pixel) is either separated from or grouped with other voxels based on key attributes (e.g., elevation, aliveness, color, slope, substance). After segmentation, each group then undergoes classification by coding each object either manually or automatically using machine learning or neural network algorithms which can achieve 70–90% classification accuracy. Geo- and marine scientists, for instance, will classify models using data that can be plotted in geographical information systems (GIS), since GIS can also be used to visualize, process and analyse these data for scientific purposes (Lim et al., 2017). Scientists thereby create meaning through interpretation of these models, but the models themselves provide the means for doing so.

VR technologies produce oceanic space through a promise for immersion, which can only be achieved if data gets produced in the ways outlined. Feeling immersed in the VR model depends upon the extent by which “a display system can deliver an inclusive, extensive, surrounding, and vivid illusion of virtual environment to a participant” (Slater & Wilbur, 1997). As a result, “virtual reality is being increasingly applied in ocean science as a tool for scientific exploration, discovery, and education” (Walcutt et al., 2019).

Nonetheless, the relationship between scientists and VR is by far the most tenuous, as the production of a VR model from the 3D model may or may not meet scientific expectations. Often, VR and augmented reality (AR) technologies are understood as technologies that focus on the production and consumption of content” (de Oliveira et al., 2022) rather than scientific analyses. They are perceived as vehicles for improving ocean education (Walcutt et al., 2019; Kasinathan et al., 2021) and for driving conservation policies (Pulido Mantas et al., 2023). As such, VR is understood as “informational,” with the promise of providing contexts for “increasing situational awareness” (de Oliveira et al., 2022) for multiple stakeholders. Immersion into VR oceanic space thus functions as a digital proxy for being in the field, which benefits scientific communication with individuals who will not be able to go to the field while simultaneously raising questions about equitable access to environments and VR technologies, as physically being in the seas, or even at its surface, can only take place during certain times and with enough resources.

**Fig. 4:**
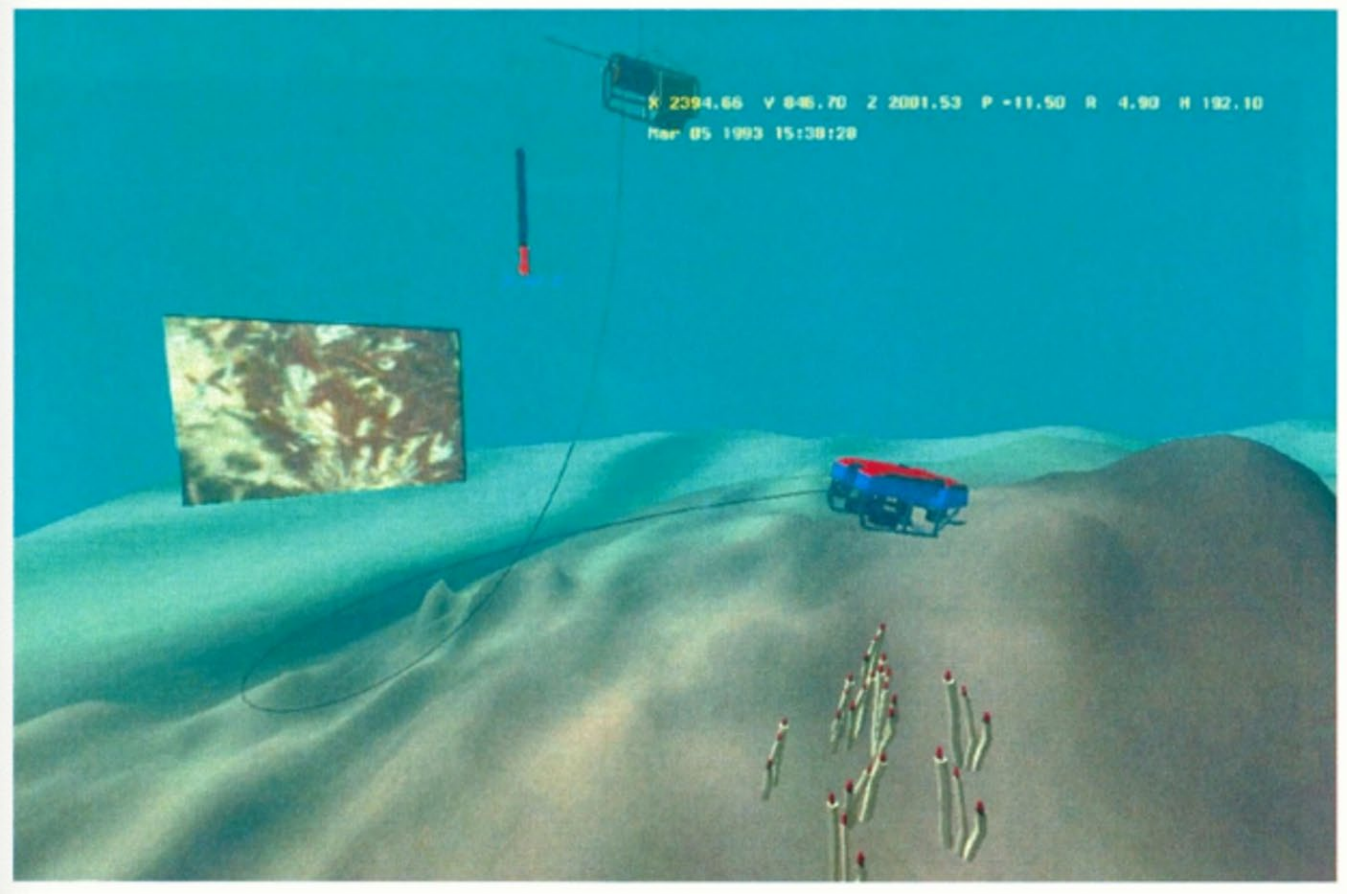
Screen shot of JASON interactive mapper (Feldman et al., 1994, pp. 256)

Experiencing immersion, however, depends on what “situation” or oceanic space gets represented—through which forms of data, which kinds of VR output devices, and where users encounter it. For example, data inputs into virtual reality environments may come from multiple sources. Early representations of oceanic space in VR relied primarily on numeric data (Fig. 4); and VR oceanic spaces may be represented as more symbolic or mimicking realism, with each style providing certain gains and limitations. For instance, realistic portrayals require 3D models with much higher resolutions, requiring more digital “space” than other approaches (de Oliveira et al., 2022). However, more “realistic” representations tend to promise greater effects for experiencing immersion, as viewers may be placed within the model and be provided with forms of interaction (Slater & Wilbur, 1997). Being able to be “inside” the representation can function to provide a more “interesting” form of learning than passive consumption of video or still images (de Oliveira et al., 2022) while interacting with the environment intends to engage sensory and cognitive forms of understanding in the user.

In sum, making oceans “virtual” emerges out of specific cultural and historic contexts that seek to assert an individualistic form of immersive sensory experience through a range of technological techniques and artifacts. As a result, VR technologies must be shaped and deployed in specific ways that deal with the ocean itself and surrounding contexts. Through this, VR technologies and practices flatten and distort the seas to give an impression of depth that can be modelled into a host of different analytic and informational forms of immersion.

## Mediating oceanic spaces: volumes, scales, and senses

From this analysis, VR technologies mediate the vertical and volumetric dimensions, scales, and sense experiences between people and oceanic spaces. They construct simplified containers, offer a multi-scalar experience (tending towards the local), and provide means for exploring other sense beyond the visual.

### Mediating verticals and volumes

The relations between scientists and technologies used to produce 3D models and VR environments of oceanic space mediate and amplify the vertical and volumetric aspect of the seas. As argued by geographers Bradley Garrett and Karen Anderson, the use of drones has given volume to the visual of spatialized knowledge (Garrett & Anderson, 2018, p. 343). They ascend into the air and descend into the sea in manners not possible for the human body. Instead of thinking oceans as surfaces to be crossed or as underwater realms of otherness, VR environments combine arid and liquid worlds, extending oceanic space from the watery seabed below to the airy and coastal environs above the sea’s surface. They also begin to incorporate marine organisms that live on the seabed, such as coral, into their representations. These environments thus add volume to understandings of oceanic space (LaViola et al., 2009) and some of its inhabitants through the mediums of air and water. In other words, volumes are treated as navigable areas by which attention focuses on surfaces such as land, sea level, and seafloor. For instance, 2D topological maps have limitations in their ability to represent vertical surfaces, such as cliffs. In 3D, such features get produced with greater legibility. This greater degree of legibility highlights desires in producing topographies that include a vertical dimension, specifically the inclusion of depth.

Adding depth assists in creating a fundamental likeness of the 3D model to the ocean floor it intends to represent. Hence, a primary ambition for these 3D models is to produce a representation that can be experienced as “real” or “truthful.” However, this cannot be done without the inclusion of additional partners in industry and elsewhere which produce the technologies relied upon to see in three dimensions (Braun, 2000; Anthony, 2020). Hence, marine scientists must deal with different domains of knowledge that permeate how scientific practices occur in each place. In most instances, these tools function as black boxes, with attention focused on what tools can produce rather than how. To assist in creating a likeness and calibrating technological errors, additional methods, such as taking seabed sediment samples, occur simultaneously with these reconstructions. So, although the 3D models are constructed to be modular to better represent a “real” space that changes over time, scientists and the VR technologies used create simplified versions to achieve models that can be interpreted or analyzed.

In other words, producing VR oceans still heavily remains fixated on surfaces, because it is intended to produce the 3D container, not necessarily what is in it. Therefore, by focusing on surfaces, VR oceanic space collapses the difference between the different volumetric and sensory properties of air and water that its virtual environs replicate. Similarly, the subterranean space remains peripheral to those organisms and technical apparatuses on top of the seabed. This focus and the volumetric navigability through VR provide the need for reference points that determine “the boundary between heights and depths” to become nearly infinite in possibility within the same model. For example, sea levels can be adjusted in VR to account for both highest and lowest depths (von Hardenberg & Mahony, 2020, p. 598). Thus, VR allows for the convergence of multiple interests and sidesteps the need for standardizing established baselines, such as mean sea level, which promote the idea of a “stable sea” (von Hardenberg, 2020, 2021). VR oceans ultimately represent a sandbox model to test out ocean features.

However, the underlying structures of data and 3D modelling invite serious considerations. One example is thinking with how the “precession of the model” will alter scientific interpretations and practices. Essentially, VR, as one form of computer modelling, provides for simulated temporal and spatial mobilities as well as the generation of data, where oceanic representation may get “undermined…broken…inverted” (Rheinberger, 2015). The very construct of plasticity, flexibility, and variability in such models points to possibilities in which it may be difficult to assess the benefits and losses generated between analyses based on simulations of future/speculative scenarios (e.g., rising sea-levels) as opposed to conditions. Additionally, the production of oceanic space in VR, along the entire way, requires that some data get used while other data get rejected. Hence, these representations must deal with structured forms of “missingness,” by which some data do not get included, usually systematically and deliberately (Mitra et al., 2023). Further inquiry into how missing data affects, rejects, and/or subverts VR models of oceanic space could help to achieve a less instrumental understanding of these areas that these technologies service. However, to call into question the seamless operation of VR models by “centralizing and politicizing” structured exclusions (Giraud, 2019) will run counter to desires for creating “digital twins” (see Tzachor et al., 2023) and creating immersive experiences for VR users.

### Mediating scales

The dimensional realities of space, including the vertical and volumetric, show to some extent why VR technologies are seen as a useful scientific application, illustrating “how science is shaped by space, and of how science has helped make the modern world.” (von Hardenberg & Mahony, 2020, p. 607). For several decades, 3D models of the seas have assisted in “improving the legibility of the world” and helping to realize “an understanding of the world as a geoengine that can be altered, modified, and engineered on a global scale” (von Hardenberg & Mahony, 2020). In this sense, it is possible to argue that representations of the seas in VR are attempting to help create an experience of the global environment (Camprubí, 2016) and/or begin to provide one way for the global environment to shape our day-to-day experience (Camprubí & Lehmann, 2018). By adding volume to representations of the oceans, VR brings a sense of the global seas nearer than before.

However, creating oceanic space in VR is often restricted to a local scale, giving it the sense of a meaningful place. Although these virtual environments are intended to function as digital replicas of actual physical locations (Lehman, 2021), they tend to represent small, site-specific scales which have yet to reach the planetary level. The scientific practices employed to generate a 3D interactive virtual environment of underwater and coastal environments, invest a great deal of time and energy, recreating a precise, local location, often focusing on resolution quality because it makes the space understandable as place. Hence, these techniques underscore the importance of place that large-scale data accumulation networks, such as the Argo program, sought to get away from (Lidström 2023, p. 125). Certainly, such large-scale concerns continue in projects like Seabed 2030 which aims to chart the world’s entire ocean floor (Seabed 2030, 2022). However, VR oceanic space reconstructions do not just give vertical depth to the seafloor but also produce volumetric space in specific locales for (possible) “immersive” experiences. VR lends possibilities for playing with the sense of scale, allowing access to inaccessible areas, or positioning avatars to take on unique perspectives (Walcutt et al., 2019). With avatars, however, the volumetric spaces must be made navigable, which either can preserve or collapse the sense of scale. VR spaces thus serve as experimental grounds for observing, adjusting, and guaranteeing the movements of matter and people across scales rather than imposing boundaries or limits upon this space (Campbell, 2019). By “securing” the volumetric space of oceans, both above and below its surface, VR works to establish means for harnessing greater control over human and nonhuman entanglement (von Hardenberg & Mahony, 2020, p. 601).

### Mediating senses

Anthropologist Stefan Helmreich argues that technologies for representing the seas have moved from the tactile (e.g., dredgers), to the auditory (e.g., acoustics), to the visual (e.g., Google Ocean) (Helmreich, 2011). Like previous technologies relying upon the visual (Deloughrey, 2014, p. 260), VR harnesses sight to recreate the seas. However, the visual in VR is not about seeing as much as it is about locating the viewer and that viewed in a spatial relationship with each other. Virtual reality applications in marine science are intended to “immerse” scientists in oceanic spaces and allow them to interact with them (Wheless et al., 1995), a move which counteracts the “weightlessness” felt in other digital models (Helmreich, 2011, p. 1235–1236). By providing sense-based simulations (albeit primarily ocular), VR oceanic spaces have the capacity to increase feelings of “spatial presence,” heightening one’s sense of actually “being there” (Steuer, 1992; Slater & Wilbur, 1997; Wirth et al., 2007). This can increase feelings of being connected to the represented environment (Breves & Heber, 2020).

Building off Helmreich, then, VR is moving beyond the visual to incorporate the sense of proprioception, or one’s awareness of the movement and position of their body, to generate a sense of presence (Bown et al., 2017). For instance, researchers report better clarity in respect to senses of scale and distance (Walcutt et al., 2019). Additionally, analysis of VR environments, such as “closed world interaction” (Lin and Loftin, 1998), suggest possibilities for altering human conceptions of the seas. Hence, 3D environments may represent a time-space compression (Harvey, 1989) between the significant “places” for doing research, the marine station and the field. VR and telepresence continue to be thought as possible formats for collaboration where “all stakeholders could access the same virtual environment…[to] aid in mission planning, task delegation, and policy making” (Walcutt et al., 2019). That said, it remains difficult to know how these incremental benefits might impact scientific analyses and broader societal conceptions of oceanic space.

The desire for an immersive experience of the oceans, not just for themselves but for others, shapes scientists, their practices, and the VR products. By attempting to mediate additional sense, such as proprioception, the scientists’ ability to interact with these digital oceanic spaces “changes, influences, and shapes the investigator” (Benson, 2015, p. 318). Early VR experimentation with the CAVE (cave automatic virtual environment) already understood that “two important aspects of virtual reality are suspension of disbelief, in which the user ignores the medium, and viewer-centered perspective, in which the perspective view is simulated from the location of the viewer” (Cruz et al., 1992). Today, continuing to create immersive virtual environments (IVEs) in which interaction with the mediated place through an avatar is seen as valuable for simulating closer connections to animals and nature (Ahn et al., 2016). However, like the humanoid robot OceanOne, avatars in VR tend to reinscribe the reproduction of human bodies and sensory perceptions. The main difference here is that marine robotics attempt to implant the human operator in the sea’s environment (Braverman, 2020, pp. 157–159) while VR attempts to bring the sensation of the environment to the viewer. The possibilities for “provid[ing] ways of viewing the data from perspectives not possible in the real world,” including from different positions, from non-human perspectives, and through the temporal dimension (Wheless et al., 1995, p. 52) still seem to be lagging in terms of oceanic spaces produced in VR by geo- and marine scientists. Perhaps, increasing collaborations with those in the humanities and the arts could assist in providing theoretical insights and analytic tools for interpreting different vantage points and avatar possibilities along with the justifications for doing so. Through further experimentation and extended forms of collaboration, being able to interact digitally with VR oceanic space may provide alternative relations for thinking and relating to the seas.

## Conclusion

To produce virtual oceanic space for VR output devices, scientists rely upon a host of VR technologies, including drones, satellite positioning systems, HD cameras, and a wide range of software programs (e.g., photogrammetry) developed by computer scientists that allow for mathematical calculations of depth based on still imagery and its visualization in 3D digital spaces. VR technologies therefore not only mediate the oceans in one’s use of them, but they also exist as products of (technological) mediations upon mediations. Furthermore, the digital depictions they create represent key changes in how oceanic space comes to be understood and potentially the lives that inhabit them. Considering that seafloor samples were initially taken to help lay underwater cables but began to be used by marine biologists (Rozwadowski, 2001, p. 243), it remains to be seen how VR oceans will further impact these fields. For the time being, oceanic space in VR can be characterized as assisting the production of local, place-based engagements, specifically through positionality and the sensation of interactivity, or immersion. There is no reason to assume that VR ocean environments need to remain local in scale, but the process for building oceanic spaces in VR still struggle with having to simplify and erase much of the oceans themselves because of the ocean’s materiality and technological limitations, which contributes to the difficulty in representing VR oceans at large scales (Ratté, 2019, p. 147).

VR technologies are therefore not neutral, given they foreground certain qualities of the ocean while backgrounding others. For instance, the application of VR technologies show a fundamental desire to adhere to the likeness of oceanic place. There is a commitment to the “reality” or “truthfulness” of the photo- and videographic image (Rose, 2022, p. 27) in VR ocean models, since photographic and videographic techniques readily serve these intentions (Tagg, 1988, 2009) perhaps even more so than numeric data. Hence, photographic images, video stills, and the 3D models built from them “image” the environment, claiming objectivity and authority (Daston & Galison, 2021). Marine science thus leverages VR technologies to further its apolitical position. Although this paper highlights that VR oceanic environments entail a careful selection of data, a plastic 3D model for interpretive flexibility, and virtual reconstructions which provide for the possibility of positioning a non-neutral avatar in these spaces, VR still seems to serve “objective” science. As Walcutt et al. claim, “Virtual reality provides a less curated experience than two-dimensional data visualization, allowing users to interact with and interpret data in a manner that is less constrained by the author’s perspective, influence, or bias” (2019). Yet, such a claim appears to miss the mark, as this article clearly demonstrates that interaction and interpretation of VR models requires as much, if not more, work than other scientific and technological methods for mediating the seas. Indeed, VR technologies both prop up and function as an entire technoscientific amalgamation that produces certain types of a possible virtual ocean among many.

## Footnotes

1 Booking appropriately sized vessels that can handle specific environmental conditions and the equipment required can be difficult and expensive.
